# Socioeconomic disparities in changes to preterm birth and stillbirth rates during the first year of the COVID-19 pandemic: a study of 21 European countries

**DOI:** 10.1093/eurpub/ckad186

**Published:** 2024-07-01

**Authors:** Jennifer Zeitlin, Marianne Philibert, Henrique Barros, Lisa Broeders, Jan Cap, Željka Draušnik, Hilde Engjom, Alex Farr, Jeanne Fresson, Miriam Gatt, Mika Gissler, Günther Heller, Jelena Isakova, Karin Källén, Theopisti Kyprianou, Marzia Loghi, Kirsten Monteath, Laust Mortensen, Tonia Rihs, Luule Sakkeus, Izabela Sikora, Katarzyna Szamotulska, Petr Velebil, Ivan Verdenik, Guy Weber, Irisa Zile, Oscar Zurriaga, Lucy Smith, Euro-Peristat Research Group and the Population Health Information Research Infrastructure, Jeannette Klimont, Alex Farr, Sophie Alexander, Marie Delnord, Judith Racapé, Gisèle Vandervelpen, Wei-Hong Zhang, Rumyana Kolarova, Evelin Jordanova, Jelena Dimnjakovic, Željka Draušnik, Urelija Rodin, Theopisti Kyprianou, Vasos Scoutellas, Jitka Jirova, Petr Velebil, Anne Vinkel Hansen, Laust Hvas Mortensen, Liili Abuladze, Luule Sakkeus, Mika Gissler, Anna Heino, Melissa Amyx, Béatrice Blondel, Anne Chantry, Catherine Deneux Tharaux, Mélanie Durox, Jeanne Fresson, Alice Hocquette, Marianne Philibert, Annick Vilain, Jennifer Zeitlin, Dimitra Bon, Günther Heller, Björn Misselwitz, Aris Antsaklis, István Sziller, Védís Helga Eiríksdóttir, Jóhanna Gunnarsdóttir, Helga Sól Ólafsdóttir, Karen Kearns, Izabela Sikora, Rosaria Boldrini, Marina Cuttini, Serena Donati, Marzia Loghi, Marilena Pappagallo, Janis Misins, Irisa Zile-Velika, Rita Gaidelyte, Jelena Isakova, Audrey Billy, Aline Lecomte, Jessica Pastore, Guy Weber, Miriam Gatt, Peter Achterberg, Lisa Broeders, Ashna Hindori-Mohangoo, Jan Nijhuis, Rupali Akerkar, Hilde Engjom, Kari Klungsøyr, Ewa Mierzejewska, Katarzyna Szamotulska, Henrique Barros, Carina Rodrigues, Mihaela-Alexandra Budianu, Alexandra Cucu, Mihai Horga, Lucian Puscasiu, Petru Sandu, Vlad Tica, Ján Cáp, Miha Lucovnik, Ivan Verdenik, Adela Recio Alcaide, María Fernández-Elorriaga, Mireia Jané, Maria José Vidal, Óscar Zurriaga, Karin Källén, Anastasia Nyman, Tonia Rihs, Diane Anderson, Samantha Clarke, Hannah McConnell, Alison Macfarlane, Sinead Magill, Kirsten Monteath, Siobhán Morgan, Joanne Murphy, Mark Piper, Sonya Scott, Lucy Smith, Craig Thomas, Martin Williams, Claudia Habl, Cara Pries, Richard Pentz, Stefan Mathis-Edenhofer, Andrea Schmidt, Alexander Grabenhofer-Eggerth, Johannes Weiss, Sophie Sagerschnig, Anita Gottlob, Lorenz Dolanski, Alexander Degelsegger-Marquez, Beate Gruber, Katharana Habimana, Petronille Bogaert, Marie Delnord, Nienke Schutte, Kim Vyncke, Tadek Krzywania, Linda Abboud, Miriam Saso, Brecht Devleesschauwer, Barthélémy Moreau de Lizoreux, Pascal Derycke, Pierre Daubresse, Sasha Milbeck, Karin De Ridder, Charles-Andrew Van de Catsyne, Sejla Cilovic Lagarija, Anina Chileva, Jelena Dimnjakovic, Jakov Vukovic, Sarka Dankova, Ondrej Májek, Sigrid Vorobjov, Jane Idavain, Merika Rätsep, Hanna Tolonen, Mari Mäkinen, Mika Gissler, Jennifer Zeitlin, Marianne Philibert, Laure Carcaillon-Bentata, Romana Haneef, Tatjana Makovski, Martin Thißen, Stefanie Seeling, Angela Fehr, Thomas Ziese, Christina Georgakopoulou, Elena Petelos, Christog Lionis, Dimitra Lingri, Tóth Kornél, Ágnes Töll, Peter Bezzegh, István Csizmadia, Róbert Láng, Kiss Csaba, Alan Cahill, Michael Courtney, Pauline White, Kelly Ailish, Patricia Clarke, Sharon Kappala, Breda Smyth, Luigi Palmieri, Brigid Unim, Andrea Faragalli, Janis Misins, Irisa Zile, Ausra Zelviene, Audronè Astrauskiené, Guy Weber, Dorita Buttigieg, Neville Calleja, Oleg Lozan, Rodica Gramme, Mariken Tijhuis, Daniela Moye Holz, Henk Hilderink, Linda Berger-Symons, Marit de Vries, Håkon Haaheim, Frode Forland, Zuzana Nordeng, Tricia Larose, Malgorzata Strozyk, Pawel Maryniak, Krystyna Drogon, Karolina Węgrzyn, Tomasz Wisniewski, Kinga Paciorek, Paulo Nogueira, Leonor Bacelar-Nicolau, Rodrigo Feteira Santos, Luís Lapão, Mariana Peyroteo, Marília Silva Paulo, Teresa Montez, Carlos Dias, Veronica Gomez, Lucinda Oliveira das Neves, Andre Peralta-Santos, Petru Sandu, Elena Gabriela Gaftonie, Edit Fekete, Lacramioara Brinduse, Silviu Radulescu, Maja Krstic, Aleksandar Medaveric, Jan Cap, Metka Zaletel, Matej Vinko, Tatjana Kofol Bric, Inmaculada León Gómez, Carmen Rodriguez-Blazquez, M João Forjaz, Marta Marin, Amparo Larrauri, Rebeca Ramis, Asuncion Diaz, Ester Angulo-Pueyo, Cesar Garriga, Teresa Valero, Francisco Estupiñán, Sandra Garcia-Armesto, Enrique Bernal-Delgado, Juan González García, Javier Gómez-Arrue Azpiazu, Ramon Launa Garces, Teresa López-Cuadrado, Ramón Launag, Carlos Telleria, Meriam Seral, Ester Angulo-Pueyo, Lovisa Syden, Ashley Akbari, Ronan Lyons, Sarag Aldridge

**Affiliations:** Obstetrical, Perinatal and Pediatric Epidemiology Research Team (EPOpé), Centre for Research in Epidemiology and Statistics (CRESS), INSERM, INRAE, Paris Cité University, Paris, France; Obstetrical, Perinatal and Pediatric Epidemiology Research Team (EPOpé), Centre for Research in Epidemiology and Statistics (CRESS), INSERM, INRAE, Paris Cité University, Paris, France; EPIUnit, University of Porto, Porto, Portugal; The Netherlands Perinatal Registry (Perined), Utrecht, Netherlands; National Health Information Center, Bratislava, Slovakia; Division of Public Health, Croatian Institute of Public Health, Zagreb, Croatia; Division of Mental and Physical Health, Norwegian Institute of Public Health, Bergen, Norway; Division of Obstetrics and Feto-Maternal Medicine, Department of Obstetrics and Gynecology, Medical University of Vienna, Vienna, Austria; Department for Research, Studies, Assessment and Statistics (DREES), French Ministry of Health, Paris, France; National Obstetric Information System, Directorate for Health Information and Research, Pieta, Malta; Department of Knowledge Brokers, THL Finnish Institute for Health and Welfare, Helsinki, Finland; Institute of Molecular Medicine and Surgery, Karolinska Institute, Stockholm, Sweden; Social Data Department, Institute for Quality Assurance and Transparency in Healthcare (IQTIG), Berlin, Germany; Health Statistics Department, Health Information Centre, Institute of Hygiene, Vilnius, Lithuania; Department of Evaluation and Analysis, Epidemiology and Methodological Support Unit, Karolinska Institute, Stockholm, Sweden; Health Monitoring Unit, Ministry of Health, Nicosia, Cyprus; Directorate for Social Statistics and Welfare, Italian Statistical Institute (ISTAT), Rome, Italy; Information Services Division, Public Health Scotland, Edinburgh, UK; Department of Child Life and Health, University of Edinburgh, Edinburgh, UK; Department of Public Health, University of Copenhagen, Copenhagen, Denmark; Denmark Statistics, Copenhagen, Denmark; Federal Statistical Office (FSO), Neuchâtel, Switzerland; Estonian Institute for Population Studies, Tallinn University, Tallinn, Estonia; The National Perinatal Reporting System, Health Pricing Office, Dublin, Ireland; Department of Epidemiology and Biostatistics, National Research Institute of Mother and Child, Warsaw, Poland; Institute for the Care of Mother and Child, Prague, Czechia; 3rd Faculty of Medicine, Charles University, Prague, Czechia; Department of Obstetrics & Gynecology—Research Unit, University Medical Centre, Ljubljana, Slovenia; Department of Epidemiology and Statistics, Directorate of Health, Luxembourg, Luxembourg; The Centre for Disease Prevention and Control of Latvia, Riga, Latvia; Public Health General Directorate, Valencia Regional Public Health Authority, Valencia, Spain; Public Health and Preventive Medicine Department, University of Valencia, Valencia, Spain; Centre for Network Biomedical Research in Epidemiology and Public Health (CIBERESP), Madrid, Spain; Department of Health Sciences, College of Life Sciences, University of Leicester, Leicester, UK

## Abstract

**Background:**

Despite concerns about worsening pregnancy outcomes resulting from healthcare restrictions, economic difficulties and increased stress during the COVID-19 pandemic, preterm birth (PTB) rates declined in some countries in 2020, while stillbirth rates appeared stable. Like other shocks, the pandemic may have exacerbated existing socioeconomic disparities in pregnancy, but this remains to be established. Our objective was to investigate changes in PTB and stillbirth by socioeconomic status (SES) in European countries.

**Methods:**

The Euro-Peristat network implemented this study within the Population Health Information Research Infrastructure (PHIRI) project. A common data model was developed to collect aggregated tables from routine birth data for 2015–2020. SES was based on mother’s educational level or area-level deprivation/maternal occupation if education was unavailable and harmonized into low, medium and high SES. Country-specific relative risks (RRs) of PTB and stillbirth for March to December 2020, adjusted for linear trends from 2015 to 2019, by SES group were pooled using random effects meta-analysis.

**Results:**

Twenty-one countries provided data on perinatal outcomes by SES. PTB declined by an average 4% in 2020 {pooled RR: 0.96 [95% confidence intervals (CIs): 0.94–0.97]} with similar estimates across all SES groups. Stillbirths rose by 5% [RR: 1.05 (95% CI: 0.99–1.10)], with increases of between 3 and 6% across the three SES groups, with overlapping confidence limits.

**Conclusions:**

PTB decreases were similar regardless of SES group, while stillbirth rates rose without marked differences between groups.

## Introduction

Most adverse pregnancy outcomes are more common among socially disadvantaged women.^1^ Lower socioeconomic status (SES) is a risk factor for a wide variety of pregnancy complications and adverse outcomes, including pre-eclampsia, foetal growth restriction and preterm delivery as well as stillbirth and neonatal death.^1–6^ The mechanisms underlying these effects are multiple and incompletely understood, but rises in stillbirth and infant mortality during economic downturns show that they are dynamic.^7^ At the onset of the COVID-19 pandemic, there was concern that pregnant women of lower SES and their babies would be most affected by the pandemic’s consequences. These concerns related to potentially greater exposure to the SARS-CoV-2 virus and more severe outcomes when these women were infected as well as to indirect effects resulting from restricted access to health care, economic hardship and increased stress and anxiety. Stress is related to preterm birth, for instance, and also more prevalent among women of lower social status.^8^^,^^9^ Research also shows that lower SES is associated with inadequate care during pregnancy and less optimal care pathways which may have increased vulnerability to healthcare restrictions during the pandemic.^1^^,^^10^

Research on pregnant women and newborns known to be infected with COVID-19 confirmed increased mortality and morbidity associated with lower SES and ethnic minority status.^11^^,^^12^ In contrast, data are scarce on possible socioeconomic disparities in the pandemic’s indirect effects. Results in the general population of pregnant women and newborns have been reassuring, belying initial concerns. Many studies have reported unexpected decreases in preterm birth (PTB) rates in 2020, while stillbirth rates appeared to remain stable. This surprising decline in PTB remains unexplained, despite a now prolific literature from hospital and country studies, which has been synthesized in several systematic reviews.^13–15^ These reviews document moderate reductions in preterm birth in high-income countries during the pandemic lockdowns of between 4 and 9% corresponding to odds ratios (OR) of 0.91 [95% confidence interval (CI):0.84–0.99], 12 studies,^13^ 0.94 (95% CI: 0.91–0.98), 28 studies^14^ and 0.96 (95% CI: 0.94–0.98), 62 studies.^15^ A recent study of population-based data from 18 countries reported a similar pooled OR of 0.96 (95% CI: 0.95–0.98).^16^ All these reviews find evidence of high heterogeneity in effects between studies and countries, which may be attributable to differences in methods or quality of the studies^17^ as well as to differences in the actual effects of lockdowns in different populations. This heterogeneity in results is also found in large population-based studies, some of which have documented decreases in preterm birth,^18–20^ while others find no change.^21^^,^^22^

There are two main hypotheses for the reduction in the preterm birth rate. First, it is possible that restricted access to healthcare limited medically indicated preterm births. This mechanism could result in suboptimal care, with potential consequences for rates of stillbirth,^23^ as many indicated preterm births are undertaken to reduce the risks of stillbirth. The second hypothesis is that lockdowns had positive effects, either because of more rest or less pollution or exposure to infections. Research up to now has reported conflicting results regarding these hypotheses, with some studies reporting changes only among indicated births,^24–26^ while many studies have found change among spontaneous births, as documented in sub-group analyses from systematic reviews.^13^^,^^14^

This research up to now has not explored changes in these outcomes by socioeconomic status, which could shed light on these mechanisms. In this study, we sought to establish whether decreases in the preterm birth rate occurred across all SES groups in European countries during the first year of the COVID-19 pandemic. We hypothesized that if the reduction were due to positive effects of the lockdown, this might be accentuated in higher SES groups where living conditions may be better and financial stress less acute, whereas restricted health care or other harmful effects might affect lower SES groups more strongly and be associated with higher stillbirth rates.

## Methods

This study is based on a federated research framework developed as part of the European Population Health Information Research Infrastructure (PHIRI) project. The PHIRI project brings together 41 partners in 30 countries to share data and expertise on the COVID-19 pandemic through a European Health Information Portal on population health. Its broader goal is to construct sustainable and reactive health information systems in Europe and promote their use for policy decision. A key component of the project is to conduct research to inform public health policies and management of the COVID-19 pandemic using a federated data model with four use cases, including one on perinatal health and perinatal health inequalities.

The perinatal health use case is implemented by the Euro-Peristat network, a collaboration between statisticians, epidemiologists and clinicians from 31 countries (27 European Union member states and Iceland, Norway, Switzerland and the United Kingdom) to assess perinatal health in Europe using a common set of 10 core and 20 recommended perinatal health indicators.^27^^,^^28^ The network began in 1999 and aims to produce and analyse robust validated data in reports and scientific publications on a regular basis for use by national, European and international stakeholders who make decisions about the health and health care of pregnant women and newborns.^27^^,^^29^ Data come from routine national data sources, including vital statistics, birth registers, hospital discharge data and routine surveys.^30^

As part of the PHIRI project, a new data collection protocol using a federated framework was implemented, involving the definition of a common data model and R-scripts that produce aggregate data tables or analytic results.^31^ Each data provider constructed a database following the specifications of the common data model and ran R programmes on their local server to generate results that were transferred to the central coordinating office (see Supplementary appendix A for contributing partners). Only anonymous data aggregated at the country level are used. Anonymous data are not covered by the General Data Protection Regulation^32^ and do not require ethics review board approval.

Data items were selected based on the Euro-Peristat core indicators augmented by items identified in a Delphi consensus process with the network members.^31^ The consistent international definitions and format of the selected data items were based on previous Euro-Peristat work. Inclusion criteria for the study were all live births and stillbirths ≥22^+0^ weeks of gestation or where gestational age (GA) was missing, with a birthweight ≥500 g. Data were collected on births from 2015 to 2020 to allow modelling of time trends in the period before the COVID-19 pandemic. For the SES analysis, we utilized data for the period March to December in each year to align with the main period of the covid pandemic in 2020. Details on the protocol for the Perinatal Health Use Case C, specifications for the common data model and R-scripts have been published^31^ and are available on the Zenodo server (see Common data model: https://zenodo.org/record/7639001, R-scripts: https://zenodo.org/records/10013399).

The two main outcomes are stillbirth and live singleton preterm birth. The stillbirth rate is defined as death before or during birth ≥22^+0^ weeks of gestation per 1000 total births. The preterm birth rate is defined as live birth <37^+0^ weeks gestation per 100 total live births. Multiple births were excluded from the preterm birth outcome, as done in most of the studies on preterm birth and COVID-19. For stillbirths, where information was available, terminations of pregnancy were excluded due to differences in policies for congenital anomaly screening and pregnancy terminations in Europe.^33^ For some countries, it was not possible to exclude terminations (Belgium, Cyprus and the Netherlands).

Across Europe, the type of measure of SES available in routine datasets varied. We collected data on maternal education when it was available, as maternal education is a good marker of socioeconomic status and has been associated with perinatal outcomes in most studies.^4^^,^^34^ Previous work in the Euro-Peristat network harmonized the coding of this variable into three groups based on the International Standard Classification of Education (ISCED—UNESCO, 1997): 1: none, primary or lower secondary (levels 0–2); 2: higher secondary (level 3); and 3: post-secondary (levels 4–6).^4^ Where mother’s education data was not available, the protocol requested the nationally used measure of area-level deprivation of the mother’s place of residence in population quintiles or the mother’s occupational class. We aggregated data on deprivation score quintiles into three groups of comparable proportions to the educational groups across most countries as: 1: most deprived (lowest 20%); 2: quintiles 2 and 3 (40%); 3: least deprived (highest 40%). Maternal occupation was provided for one country (Ireland) and was grouped as: 1: most deprived (no occupation), 2: middle SES (skilled/unskilled workers or technicians/clerical/service occupations), 3: least deprived (managers/professionals), in line with local classifications.

### Missing data

There were variable rates of missing data on socioeconomic variables. The range between countries was from 0 to 14% for live births, from 0 to 33% for preterm births and from 0 to 67% for stillbirths (Supplementary table S1). We assumed data to be missing completely at random and imputed missing data based on the observed distributions for births with and without the outcomes, i.e. for preterm birth, stillbirths and live births separately. For Croatia, data on preterm birth could not be used because of an anomaly in the recording of gestational age in 2016. For Poland, data on stillbirths were only available starting in 2018, so we could not estimate trends over time.

### Statistical analysis

We described the availability of the three SES measures for all births across the countries in the Euro-Peristat study and our pregnancy adverse outcomes for the period March to December over the 6 years of data included in the study (2015–2020). For the subset of countries with SES, we computed the estimated change in preterm birth and stillbirth rates for the period March to December 2020 compared to previous years (2015–2019). We estimated country-specific relative risks (RRs) for the period March to December 2020, adjusted for linear trends for this same period in previous years (2015–2019) using binomial models. These RR estimate the deviation from the expected linear trends. We assumed linearity because of the progressive evolution in population characteristics that influence preterm and stillbirth risks and the short time period. We then reran all our models in each SES group, computing the RR for women classified into low, medium and high SES groups. We estimated RR as opposed to risk differences to compare our results with the existing literature that has used RR or OR.^13–15^

The estimated RR from these regressions and their standard errors were then synthesized using random effects meta-analyses with sub-group analyses to compare pooled effects across SES groups. We undertook a sub-group analysis restricted to large countries with decreases in preterm birth. The rationale for this analysis was to focus on countries with less variation in estimates due to larger numbers of births and because corroborating studies on decreasing preterm birth rates during the COVID-19 pandemic, using multiple methodological approaches, exist for many of them.^18^^,^^19^^,^^35^^,^^36^ Finally, we undertook a sensitivity analysis by computing pooled estimates by different type of SES measure.

We used R^®^ software (R Foundation for Statistical Computing, Vienna, Austria), version 4.1.1 (2021-08-10) with the *metafor* package for the meta-analyses.

## Results

Out of 29 countries participating in the project, 21 countries were able to provide information on socioeconomic status and adverse pregnancy outcomes. Data were available separately for three nations: Northern Ireland, Scotland, Wales and for the whole of the UK combined (including England). Eight countries (Finland, Germany, Hungary, Iceland, Norway, Romania, Sweden and Switzerland) contributed data to the PHIRI project, but did not have access to linked SES information. In Finland, Norway and Sweden, socioeconomic variables can be linked to the birth registry as part of specific research projects, but this is not done routinely for surveillance.

Of the countries with SES, 17 provided maternal education, three area-based SES and one occupation (table 1). During the period March to December over the 6 study years, more than 16 million births occurred in the 21 countries. Live singleton preterm birth rates ranged from 4.2% to 8.3%, while stillbirth rates at 22 weeks of GA and over ranged from 2.9 to 5.2 per 1000 total births. Table 1 presents the estimates of the change in preterm birth and stillbirth rates from March to December 2020 calculated as the RR of the observed to expected values taking into consideration trends over the previous 5 years. On average, the preterm birth rate from March to December 2020 was 4% lower than expected in the same period in previous years, based on the overall pooled RR of 0.96 with a 95% confidence interval (CI) of 0.94–0.97 (see Supplementary figures S1 and S2 for meta-analyses of overall estimates). Individual country estimates of the RR were heterogeneous (*I*^2^ = 66.0%) and ranged from <0.9 in Portugal to estimated increases of over 1.02 in Estonia and Malta. For stillbirth, the estimated change in 2020 was an increase of 5% [Pooled RR: 1.05 (95% CI, 0.99–1.10)] with the RR ranging from <0.7 in Cyprus to over 1.40 in Slovenia (*I*^2^ = 51.7%). In sensitivity analyses, the range of outcomes were similar in countries reporting on maternal education and those reporting using area-based measures (Supplementary figures S1 and S2).

**Table 1:** Number of births, rates of preterm singleton birth and stillbirth and overall estimated change in preterm birth and stillbirth rates for the periods March to December from 2015 to 2020 in European countries ordered by maternal SES measure

Country	Total number of births 2015–2020 (*N*)	Preterm singleton rate (%)	Observed vs. expected rate March–December 2020^a^ (RR (95% CI))	Stillbirth rate ≥22 weeks of GA (per 1000 births) (‰)	Observed vs. expected rate in March–December 2020^a^ (RR (95% CI))
Education^b^					
Austria	430 524	5.9	0.97 (0.93–1.01)	3.3	1.31 (1.09–1.57)
Belgium	592 226	6.3	0.90 (0.87–0.93)	4.7	0.84 (0.74–0.97)
Croatia^c^	182 712	5.1	—	4.5	0.89 (0.69–1.15)
Cyprus	48 661	8.3	0.95 (0.85–1.06)	4.5	0.69 (0.44–1.08)
Czechia	556 852	5.8	0.96 (0.93–1.00)	3.5	1.19 (1.02–1.39)
Denmark	308 894	4.9	0.97 (0.92–1.03)	3.1	1.04 (0.83–1.31)
Estonia	68 700	4.3	1.04 (0.91–1.18)	2.9	1.10 (0.64–1.86)
Italy	2 251 679	5.7	0.94 (0.92–0.96)	3.5	1.11 (1.03–1.21)
Latvia	101 192	4.5	0.98 (0.88–1.09)	5.2	1.12 (0.82–1.53)
Lithuania	134 963	4.2	1.02 (0.92–1.12)	4.1	0.90 (0.66–1.24)
Luxembourg	36 001	5.5	0.99 (0.85–1.16)	4.1	0.70 (0.38–1.28)
Malta	22 562	5.5	1.03 (0.85–1.26)	4.9	1.57 (0.82–2.97)
Poland^d^	928 540	5.7	0.98 (0.96–1.00)		
Portugal	437 424	6.0	0.88 (0.84–0.92)	3.2	0.94 (0.78–1.13)
Slovakia	289 669	5.9	0.97 (0.92–1.03)	5.3	1.21 (1.02–1.45)
Slovenia	99 510	5.4	1.00 (0.91–1.10)	3.0	1.41 (0.94–2.11)
Spain	1 697 808	5.6	0.96 (0.94–0.98)	3.1	1.02 (0.92–1.13)
Area deprivation					
France	3 673 222	5.4	0.95 (0.93–0.96)	5.0	1.02 (0.97–1.08)
Netherlands	837 299	5.2	1.01 (0.98–1.04)	4.7	1.05 (0.94–1.17)
UK	3 630 284	6.1	0.94 (0.92–0.95)	4.4	1.00 (0.95–1.06)
UK: N. Ireland	117 325	5.7	0.87 (0.80–0.95)	4.2	1.05 (0.77–1.44)
UK: Scotland	258 204	6.6	0.92 (0.87–0.97)	4.1	1.13 (0.90–1.40)
UK: Wales	146 860	6.4	0.93 (0.87–0.99)	4.2	0.91 (0.71–1.16)
Occupation					
Ireland	310 318	4.8	0.93 (0.88–0.99)	4.1	1.17 (0.96–1.42)

a Calculated as the relative risk (RR) of the observed to expected values taking into consideration trends over the previous 5 years.

b Mother’s educational level was the preferred variable, followed by deprivation scores and then mothers’ occupation, if several variables were available.

c Croatian data are not included in the trend analysis because of an anomaly in the recording of gestational age in 2016.

d For Poland, total births are for 2018–2020 because no registration of stillbirths in 2015–2017.

**Figure 1:**
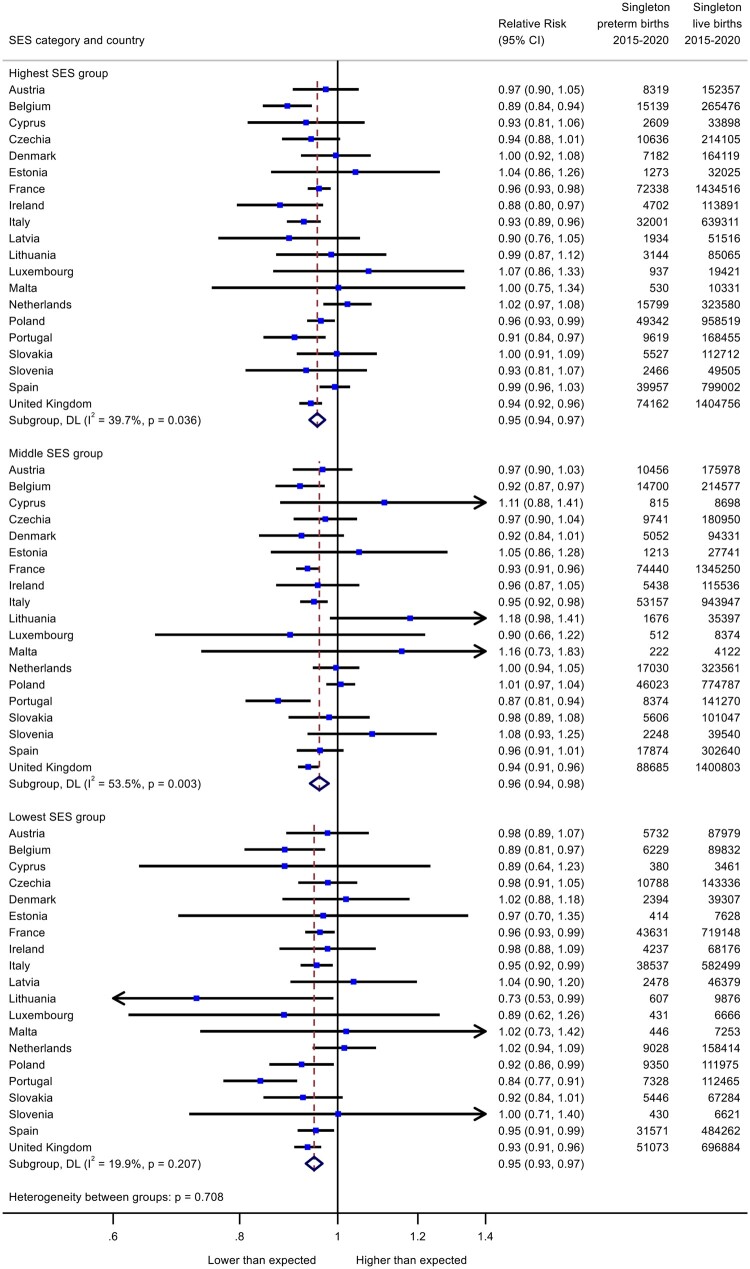
Change in preterm birth rates in March to December 2020 as measured by observed over expected risks for high, middle and low socioeconomic groups

**Figure 2:**
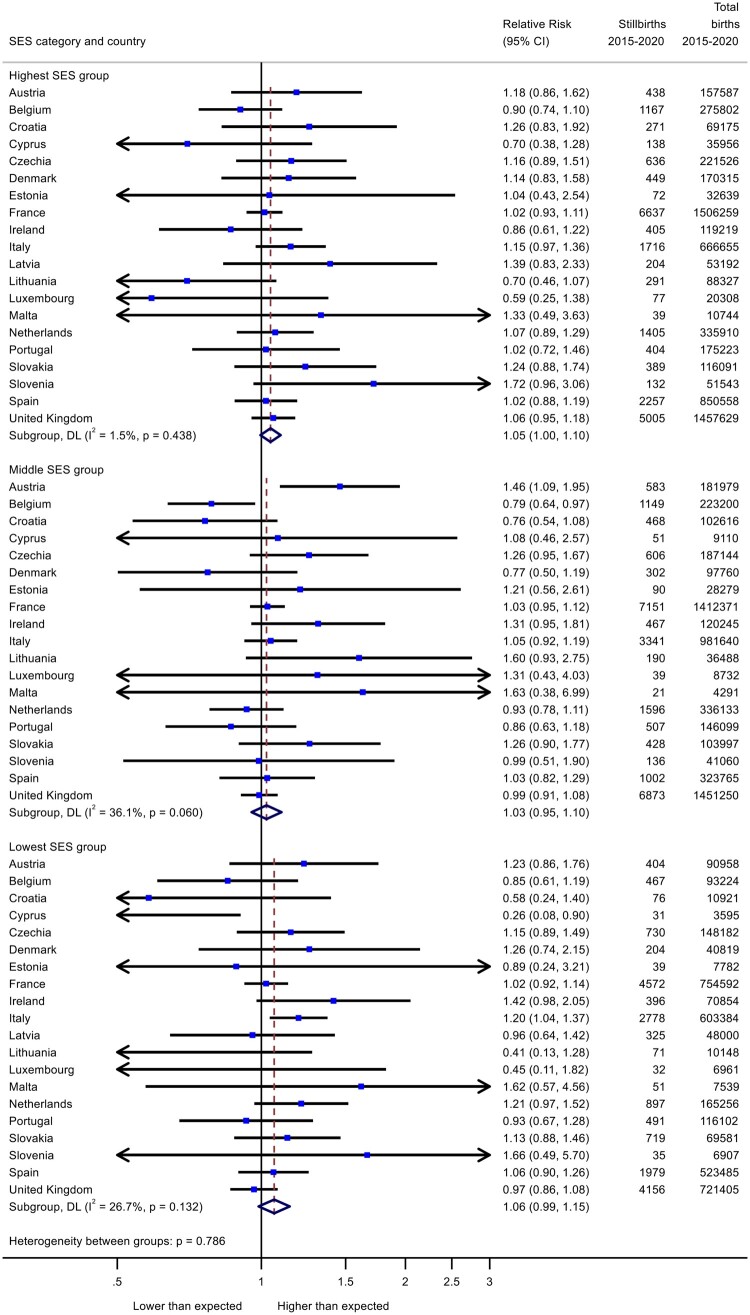
Change in stillbirth rates in March to December 2020 as measured by observed over expected risks for high, middle and low socioeconomic groups

**Figure 3:**
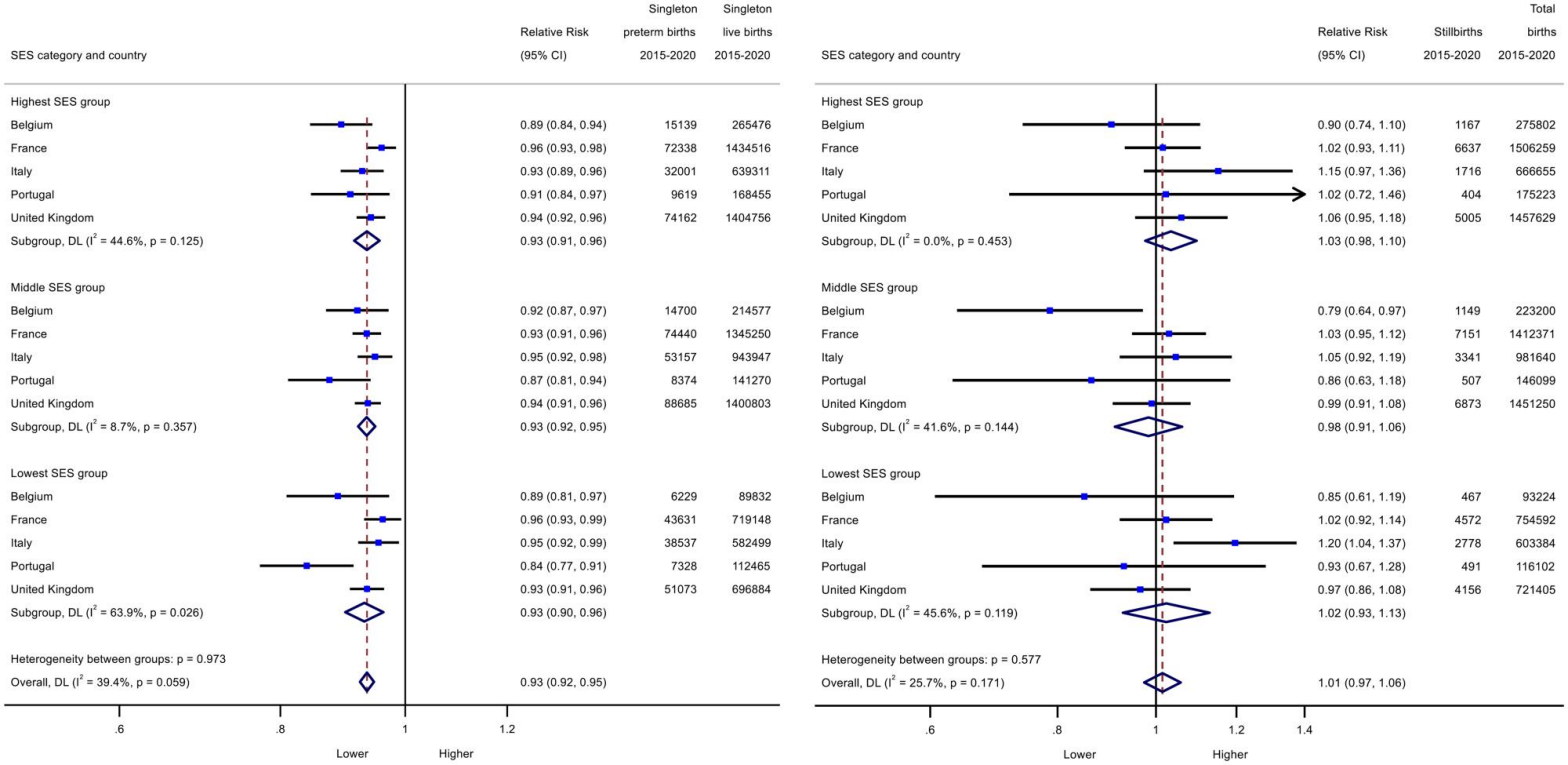
Change in preterm birth (**A**) and stillbirth (**B**) rates in March to December 2020 as measured by observed over expected risks for high, middle and low socioeconomic groups in large countries with preterm birth rate reductions

The trends in preterm birth were similar for all SES groups (figure 1), with a pooled RR of 0.95 (95% CI: 0.94–0.97) in the high group, 0.96 (95% CI: 0.94–0.98) in the medium group and 0.95 (95% CI: 0.93–0.97) in the low group. For stillbirth (figure 2), stillbirth rates increased by an estimated 6% in the low SES groups [RR = 1.06; (95% CI: 0.99–1.15)] compared to 3-5% in higher groups [medium SES RR = 1.03 (95% CI: 0.95–1.10); highest SES RR = 1.05 (95% CI: 1.00–1.10)]. Sensitivity analyses focusing on the five larger population countries experiencing substantial reductions in preterm birth in 2020 (Belgium, France, Italy, Portugal and UK), found similar pooled effects for all three SES groups (figure 3). In these countries, stillbirth rates showed a smaller increase than in the full analysis of all countries (RR = 1.01, 95% CI: 0.97–1.06). There was no clear gradient in stillbirth rates in these countries.

## Discussion

In this study of 21 European countries, we found that preterm birth rates were on average 4% lower in 2020 compared to expected trends from the previous 5 years. This decrease was the same for the highest, middle and lowest SES groups. On the other hand, stillbirth rates were 5% higher than expected in 2020 with no clear gradient across SES groups. Similar results for preterm birth were obtained in sub-group analysis restricted to the five largest population countries with substantial preterm birth reductions, but stillbirth rates did not increase.

Strengths of this study are the inclusion of 21 countries with population-based pregnancy outcome data by SES group collected for 6 years using a common protocol. Limitations are the use of different measures of SES because of lack of homogenous data. In addition, even when it was possible to use the same indicator, SES groups differ between countries because of variations in educational systems and deprivation score composition or scale. Nonetheless, in all countries the three SES groups reflect a hierarchy going from low to high and these definitions have been used in previous studies to investigate socioeconomic disparities in health.^4^^,^^34^ We also only had data for the full period from March to December by SES status, allowing us to provide an estimate of the pandemic’s impact in 2020, but not to investigate the temporal specificity of these effects. Our study used aggregated data, and we could not adjust on individual characteristics, such as maternal age, parity, smoking or body mass index. However, we assume that any changes in the characteristics of the childbearing population over time would be captured in the temporal trends over the 5-year period preceding the pandemic. Finally, while the pandemic may have affected fertility, these effects would be minimal over the time period considered, potentially affecting births at the end of the year.

The pooled measure of change in preterm birth in this study corroborate results from meta-analyses of single centre and population-based studies in high-income countries during the pandemic lockdowns in 2020.^13–15^^,^^27^^,^^29^ The added-value of this study is the finding that these changes in preterm birth were similar for high, middle and low SES groups. Based on previous literature, we hypothesized that positive lockdown effects might be more pronounced for women of higher SES, while healthcare restrictions may have had more negative effects among women of lower SES. However, our results suggest that the underlying mechanism for the change in preterm birth was similar in all groups. It is noteworthy that despite observed increases in stress and anxiety during the pandemic, which were more marked when social risk factors were present,^37^^,^^38^ there was not a detectable population effect on preterm birth even in the lowest SES group. This indicates a need to re-evaluate the stress–preterm birth pathway, in particular for acute stressors.

In contrast to preterm birth, our data indicated an overall increase in stillbirth rates. The literature on changes in stillbirth during the pandemic is less robust than on preterm birth because stillbirth is an uncommon outcome requiring studies with large sample sizes. The recent population study by the international Perinatal Outcomes in the Pandemic (iPOP) collaboration concluded that there was no change in stillbirth rates,^16^ while this has also been the conclusion of national studies in France and Italy.^18^^,^^19^ However, other reviews have concluded that stillbirths rose in high-income countries.^13^ Of note, the larger countries with decreased preterm birth rates did not have higher stillbirths rates. Different patterns for preterm birth and stillbirth changes may suggest distinct underlying pathways through which the pandemic affected these indicators. For stillbirth—in addition to indirect effects—the increased stillbirth risk may reflect risks associated with COVID-19 infection as research showed increased stillbirth risk among infected women.^38^

As in previous research,^27^^,^^29^ there was high heterogeneity in the changes observed in both outcomes across countries, raising questions about the possible role of country-level characteristics in moderating these effects. Possible moderators are magnitude of socioeconomic variation within the country, overall risks of preterm birth and stillbirth and COVID-19 related features, such as infection patterns or societal mitigation policies. This is an important area for further investigation and comparative case studies in countries with contrasting experiences could allow generation of specific hypotheses that could be tested empirically using population data.

The COVID-19 pandemic represents an important opportunity for countries to take stock and identify the improvements to routine data needed for effective surveillance and policy evalution.^39^ About one-third of countries could not provide data on key pregnancy outcomes by SES. The ability to monitor social health disparities is essential for health policy and planning, especially during crises such as pandemics, where vulnerable populations may be most affected. In some countries, SES is not recorded in routine data, while in others further linkage is required (Finland, Norway and Sweden). These latter countries may want to consider routine linkage to reduce barriers to evaluations of SES effects. In countries without SES, area-based measures may be easier to implement as geographic identifiers are often already integrated into routine sources in contrast with information on individual socioeconomic characteristics. Ideally, data would be available on individual and area-based indicators, as these capture different dimensions of social disadvantage.^40^ The new PHIRI data collection protocol piloted in this study has the advantage of allowing rapid updates to data collected at the European level, including adding and harmonizing variables. Extending and improving this federated approach will strengthen European capacity to monitor social inequalities in perinatal health and to compile and analyse data in a future pandemic.

In conclusion, this study of 21 European countries including over 16 million births in Europe confirmed slight reductions in preterm birth in many countries in 2020, with a similar effect for women regardless of their SES, whereas stillbirth rates rose with no clear gradient by SES. This study illustrates the importance as well as the feasibility of routine reporting to assess social disparities in perinatal health in Europe and can be used to guide improvements to information systems.

## Supplementary Material

ckad186_Supplementary_Data

## Data Availability

Data that supports this article are publicly available upon publication of your article upon request at the main author.

## Disclaimer

The content of this manuscript represents the views of the author only and is his/her sole responsibility. The European Research Executive Agency (REA) and the European Commission are not responsible for any use that may be made of the information it contains.

*Conflicts of interest*. None declared.
Key points

- During the first year of the COVID-19 pandemic in Europe, preterm birth rates declined in some countries, with similar decreases in all SES groups.
- In contrast, stillbirths rose slightly with variable changes across SES groups.
- Having socioeconomic indicators in routine birth data is critical for capturing the full impact of health crises, such as COVID-19.
