# Supplementary material for: Socioeconomic disparities in changes to preterm birth and stillbirth rates during the first year of the COVID-19 pandemic: a study of 21 European countries
Source: Eur J Public Health. 2024 Jul 1;34(Suppl 1):i58–66. doi: 10.1093/eurpub/ckad186 (PMC11215324; doi:10.1093/eurpub/ckad186)
Supplement: ckad186_Supplementary_Data [file ckad186_supplementary_data.zip › ejph-2023-06-phis-0326-File007.docx]

**Supplemental Table 1. Number of births and percentage of missing SES data for stillbirths, live births, singleton births and singleton preterm births for period March to December 2015-2020**

|  | Births (n) | | | | % births missing SES group | | | |
| --- | --- | --- | --- | --- | --- | --- | --- | --- |
| COUNTRY | Live births | Stillbirths | Singletons preterm | Singleton live births | Live births | Stillbirths | Singletons preterm | Singleton live births |
| Austria | 429 099 | 1 426 | 24 509 | 416 313 | 0,7% | 0,6% | 0,8% | 0,7% |
| Belgium | 589 442 | 2 782 | 36 067 | 569 883 | 14,2% | 66,9% | 15,7% | 14,2% |
| Croatia | 181 897 | 816 | 8 917 | 175 874 | 30,3% | 35,5% | 33,1% | 30,2% |
| Cyprus | 48 440 | 220 | 3 804 | 46 056 | 0,5% | 0,5% | 0,4% | 0,6% |
| Czechia | 554 881 | 1 975 | 31 165 | 538 390 | 12,1% | 20,4% | 15,6% | 11,9% |
| Denmark | 307 938 | 956 | 14 630 | 297 757 | 5,0% | 6,5% | 4,7% | 5,0% |
| Estonia | 68 499 | 201 | 2 900 | 67 394 | 0,0% | 0,0% | 0,1% | 0,0% |
| France | 3 654 860 | 18 369 | 190 405 | 3 498 908 | 2,6% | 4,1% | 3,3% | 2,6% |
| Ireland | 309 048 | 1 268 | 14 377 | 297 603 | 4,9% | 8,7% | 5,9% | 5,0% |
| Italy | 2 243 843 | 7 836 | 123 694 | 2 165 759 | 2,3% | 4,1% | 2,5% | 2,3% |
| Latvia | 100 663 | 529 | 4 412 | 97 895 | 0,0% | 0,0% | 0,0% | 0,0% |
| Lithuania | 134 411 | 552 | 5 427 | 130 338 | 6,0% | 2,9% | 5,2% | 6,0% |
| Luxembourg | 35 851 | 147 | 1 880 | 34 463 | 6,9% | 13,6% | 9,4% | 6,9% |
| Malta | 22 464 | 98 | 1 198 | 21 706 | 7,5% | 19,4% | 10,8% | 7,6% |
| Netherlands | 833 395 | 3 897 | 41 854 | 805 558 | 0,5% | 0,7% | 0,6% | 0,5% |
| Poland | 1 894 866 | 3 110 | 104 715 | 1 845 281 | 2,4% | 4,3% | 3,0% | 2,4% |
| Portugal | 436 020 | 1 401 | 25 320 | 422 192 | 6,0% | 45,1% | 4,7% | 6,0% |
| Slovakia | 288 134 | 1 536 | 16 580 | 281 043 | 12,2% | 12,8% | 13,2% | 12,1% |
| Slovenia | 99 206 | 304 | 5 143 | 95 665 | 3,3% | 4,9% | 3,5% | 3,4% |
| Spain | 1 692 573 | 5 238 | 89 403 | 1 585 903 | 9,1% | 17,1% | 9,7% | 8,3% |
| UK: MBRRACCE | 3 614 244 | 16 039 | 213 916 | 3 502 442 | 0,4% | 0,4% | 0,4% | 0,4% |
| UK: Northern Ireland | 116 828 | 495 | 6 485 | 113 233 | 0,2% | 0,8% | 0,1% | 0,2% |
| UK: Scotland | 257 156 | 1 053 | 16 342 | 249 168 | 0,2% | 0,6% | 0,3% | 0,2% |
| UK: Wales | 146 240 | 623 | 7 674 | 120 101 | 0,8% | 0,7% | 0,9% | 0,7% |
