# Supplementary material for: Socioeconomic disparities in changes to preterm birth and stillbirth rates during the first year of the COVID-19 pandemic: a study of 21 European countries
Source: Eur J Public Health. 2024 Jul 1;34(Suppl 1):i58–66. doi: 10.1093/eurpub/ckad186 (PMC11215324; doi:10.1093/eurpub/ckad186)
Supplement: ckad186_Supplementary_Data [file ckad186_supplementary_data.zip › ejph-2023-06-phis-0326-File008.pdf]

Figure S1. Change in preterm birth rates in March to December 2020 as measured by observed over expected risks by type of SES measure

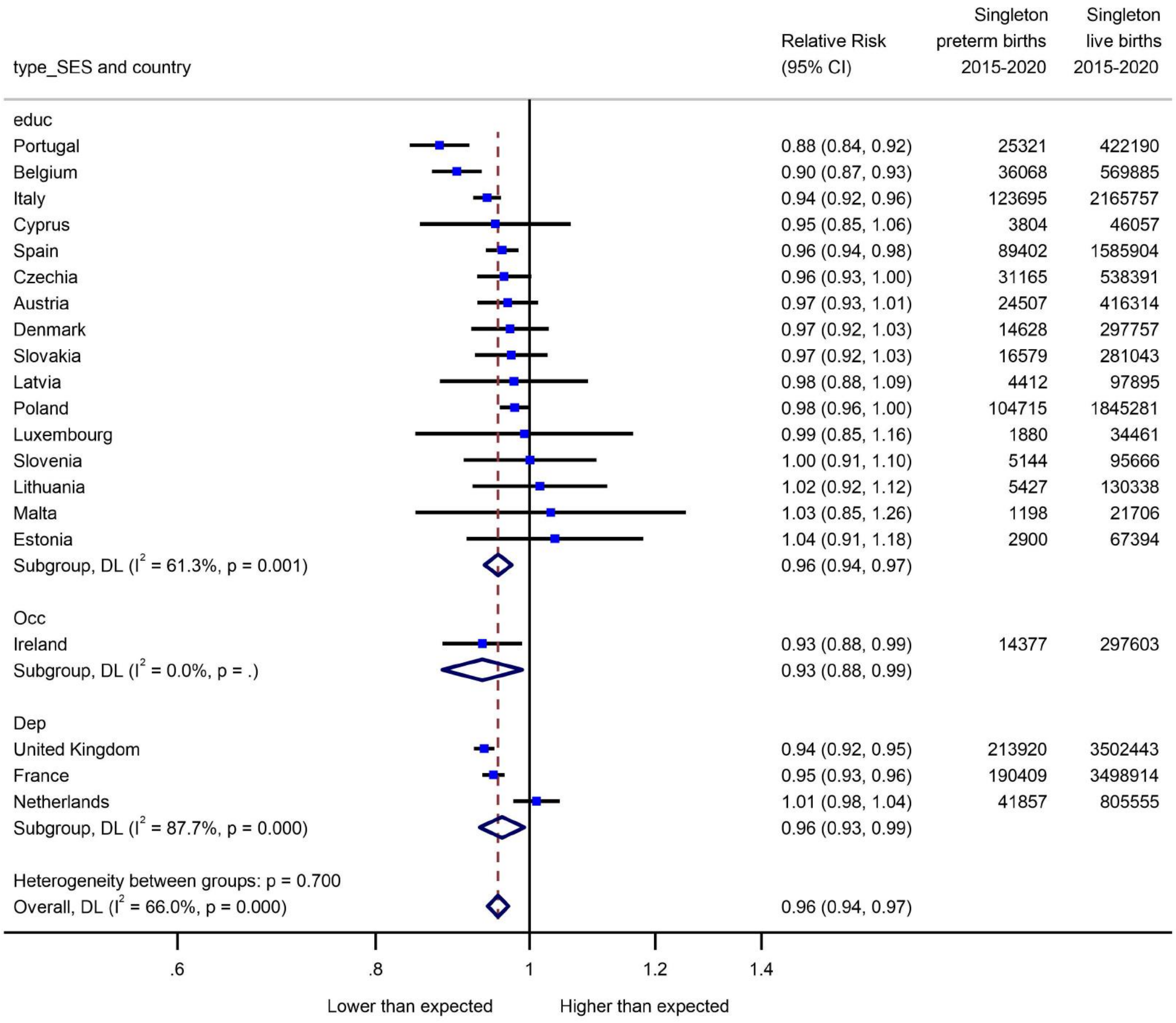

NOTE: educ: maternal education, dep: deprivation score, occ: maternal occupation
