# Supplementary material for: Socioeconomic disparities in changes to preterm birth and stillbirth rates during the first year of the COVID-19 pandemic: a study of 21 European countries
Source: Eur J Public Health. 2024 Jul 1;34(Suppl 1):i58–66. doi: 10.1093/eurpub/ckad186 (PMC11215324; doi:10.1093/eurpub/ckad186)
Supplement: ckad186_Supplementary_Data [file ckad186_supplementary_data.zip › ejph-2023-06-phis-0326-File006.docx]

**APPENDIX A.** List of data sources and data providers to the Euro-Peristat data collection 2015-2020

|  | Data sources | Data providers |
| --- | --- | --- |
| Austria | * Birth statistics (Statistics Austria) * Cause of death statistics (Statistics Austria) | * Jeanette Klimont/Statistics Austria |
| Belgium | * Vital Statistics, Statistics Belgium (Statbel) | * Gisele Vandervelpen/Statbel |
| Bulgaria | * Vital Statistics (National Statistics Institute) * National birth register (National Center for Public Health and Analysis) |  |
| Croatia | * Croatian Medical Birth Database (Croatian Public Health Institute),  * Croatian Mortality Database (Croatian Central Bureau of Statistics) - | * Željka Draušnik/Croatian Institute of Public Health |
| Cyprus | * Medical Birth register (Health Monitoring Unit, Cyprus Ministry of Health) * Causes of Death register (Health Monitoring Unit, Cyprus Ministry of Health) * Database for COVID-19 confirmed cases and deaths (Health Monitoring Unit, Cyprus Ministry of Health) | * Theopisti Kyprianou/Health Monitoring Unit, Ministry of Health |
| Czech Republic | * Institute of Health Statistics and Information of the Czech Republic (national birth register (mothers and newborns) collecting individual perinatal data.) | * Jitka Jirova/Institute of Health Information and Statistics of the Czech Republic |
| Denmark | * Medical birth register (The Danish Data authority, Danish Ministry of Health) * National patient register (The Danish Data authority, Danish Ministry of Health) * Danish causes of death register (The Danish Data authority, Danish Ministry of Health) * The Centralized Civil Register | * Anne Vinkel Hansen/ Statistics Denmark |
| Estonia | * Estonian Medical Birth Register (National Institute for Public Health) was linked with data from * Estonian Cause of Death Register (National Institute for Public Health) | * Liili Abuladze/Estonian Institute for Population Studies, Tallinn University |
| Finland | * Medical Birth Register (Finnish Institute for Health Welfare) linked with Central Population Register (Digital and Population Data Services Agency) and Cause of Death Register (Statistics Finland)  * Register on Induced Abortions (Finnish Institute for Health Welfare) for late terminations 22-24 weeks | * Mika Gissler/Finnish Institute for Health and Welfare, Helsinki (THL) |
| France^2^ | * PMSI (ATIH: Technical agency of hospitalization information) | * Annick Vilain/Department for Research, Studies, Assessment and Statistics (DREES), French Ministry of Health |
| Germany | * IQTIG (Federal Institute for the Quality of Medical Care) | *Guenther Heller/IQTIG |
| Hungary | *Hungarian Central Statistical Office (KSH)  *Hungarian National Obstetric Register | *Istvan Sziller/National Directory for Hospital Management and Andrea Valek/Semmelweis University |
| Iceland | * The Icelandic Birth Registration * Hospital register (National University Hospital) | *Helga Sol/National University Hospital |
| Ireland | *National Perinatal Reporting System (the Healthcare Pricing Office) | * Karen Kearns /Healthcare Pricing Office |
| Italy | * Birth certificates (Ministry of Health)  * Causes of deaths (Istat)  * Terminations of pregnancies (Istat)  * Miscarriages (Istat) | * Marzia Loghi / Italian National Institute for Statistics-ISTAT |
| Latvia | * Newborn Register of Latvia (Centre for Disease Prevention and Control of Latvia) * Register of Causes of Death (Centre for Disease Prevention and Control of Latvia) | * Irisa Zile / The Centre for Disease Prevention and Control of Latvia |
| Lithuania | * Medical Date of Births (Institute of Hygiene Health Information Centre) * Database of the Demographic Statistics (Central Statistical Office) * Causes of Death register (Institute of Hygiene Health Information Centre) | * Jelena Isakova / Institute of Hygiene, Health Information Centre |
| Luxembourg | * Perinatal Health Monitoring System (Luxembourg Institute of Health)  * National Causes of Death Registry (Directorate of Health of Luxembourg) | * Audrey Billy / Department of Precision Health, Luxembourg Institute of Health  * Aline Lecomte / Department of Precision Health, Luxembourg Institute of Health  * Jessica Pastore / Department of Precision Health, Luxembourg Institute of Health  * Guy Weber / Directorate of Health of Luxembourg |
| Malta | * National Obstetrics Information System (Directorate for Health Information and Research) * National Mortality Register (Directorate for Health Information and Research) | * Miriam Gatt / Directorate for Health Information and Research |
| Netherlands | * Perined (The Netherlands Perinatal Registry) | * Lisa Broeders / Perined |
| Norway | * Medical Birth Register of Norway (The Norvegian Institute of Public Health) | * Rupali Akerkar, Kari Klungsøyr/ The Norwegian Institute of Public Health |
| Poland | * Central Statistical Office * Ministry of Health | * Katarzyna Szamotulska/ National Research Institute of Mother and Child |
| Portugal | * Instituto Nacional de Estatística – Portugal (Statistics Portugal)  * Central Administration of the Health System | * Carina Rodrigues / Institute of Public Health of the University of Porto |
| Romania | * National Institute for Public Health Romania |  |
| Slovakia | *National Health Information Center | *Ján Čáp /nathinal Health Information Center |
| Slovenia | *Perinatal information system (National institute of public health) | * Ivan Verdenik / University Medical Centre, Research Unit |
| Spain | * Vital Statistics (National Statistics Office)  * Specialized Care Registry - Minimum Basic Data Set (Ministry of Health) | * Adela Recio Alcaide/ Senior Statistical State Corps and Oscar Zurriaga/ Public Health and Addictions Directorate, Generalitat Valenciana |
| Sweden | * Medical Birth Register (The National Board of Health and Welfare) | * Karin Kallen / The National Board of Health and Welfare |
| Switzerland | * BEVNAT, statistics of natural population change - vital statistics (Swiss federal Statistical Office) | * Tonia Rihs / Swiss Federal Statistical Office |
| UK, Northern Ireland | * Northern Ireland Maternity System - NIMATS (Department of Health) | * Joanne Murphy and Diane Anderson / Northern Ireland Maternal And Child Health (NIMACH) |
| UK, Scotland | * Scottish Morbidity Record 02 (maternity hospital discharge record) * National Records of Scotland Stillbirth, live birth, and infant death registrations (statutory vital event registration) | * Kirsten Monteath / Public Health Scotland |
| UK, England and Wales | *UK, Office for National Statistics (Live birth and stillbirth registration in England and Wales, notification of births in England and Wales) | * Hannah McConnell/ Office for National Statistics |
| UK, England | *Maternity Hospital Episode Statistics | *Craig Thomas / NHS Digital |
| UK, Wales | *Digital Health and Care Wales | *Mark Piper /Digital Health and Care Wales*(*DHCW*)* |
| UK, all | * MBRRACE UK (University of Oxford and University of Leicester) | * Lucy Smith / University of Leicester, MBRRACE-UK collaboration |
